# Increased phosphorylation of eIF2α in chronic myeloid leukemia cells stimulates secretion of matrix modifying enzymes

**DOI:** 10.18632/oncotarget.12941

**Published:** 2016-10-27

**Authors:** Paulina Podszywalow-Bartnicka, Anna Cmoch, Magdalena Wolczyk, Lukasz Bugajski, Marta Tkaczyk, Michal Dadlez, Margaret Nieborowska-Skorska, Antonis E. Koromilas, Tomasz Skorski, Katarzyna Piwocka

**Affiliations:** ^1^ Laboratory of Cytometry, Nencki Institute of Experimental Biology of Polish Academy of Sciences, Warsaw, Poland; ^2^ Laboratory of Lipid Biochemistry, Department of Biochemistry, Nencki Institute of Experimental Biology of Polish Academy of Sciences, Warsaw, Poland; ^3^ Laboratory of Mass Spectrometry, Institute of Biochemistry and Biophysics, Polish Academy of Sciences, Warsaw, Poland; ^4^ Department of Microbiology and Immunology, Temple University School of Medicine, Philadelphia, USA; ^5^ Lady Davis Institute for Medical Research, McGill University, Sir Mortimer B. Davis-Jewish General Hospital, Montreal, Quebec, Canada; ^6^ Department of Oncology, Faculty of Medicine, McGill University, Montreal, Quebec, Canada

**Keywords:** CML, eIF2α, ATF4, proteases, cell invasion

## Abstract

Recent studies underscore the role of the microenvironment in therapy resistance of chronic myeloid leukemia (CML) cells and leukemia progression. We previously showed that sustained mild activation of endoplasmic reticulum (ER) stress in CML cells supports their survival and resistance to chemotherapy. We now demonstrate, using dominant negative non-phosphorylable mutant of eukaryotic initiation factor 2 α subunit (eIF2α), that phosphorylation of eIF2α (eIF2α-P), which is a hallmark of ER stress in CML cells, substantially enhances their invasive potential and modifies their ability to secrete extracellular components, including the matrix-modifying enzymes cathepsins and matrix metalloproteinases. These changes are dependent on the induction of activating transcription factor-4 (ATF4) and facilitate extracellular matrix degradation by CML cells. Conditioned media from CML cells with constitutive activation of the eIF2α-P/ATF4 pathway induces invasiveness of bone marrow stromal fibroblasts, suggesting that eIF2α-P may be important for extracellular matrix remodeling and thus leukemia cells-stroma interactions. Our data show that activation of stress response in CML cells may contribute to the disruption of bone marrow niche components by cancer cells and in this way support CML progression.

## INTRODUCTION

The bone marrow microenvironment creates a unique niche, which is essential for hematopoiesis, but also supports the development of leukemia. Bone marrow stromal fibroblasts support the proliferation of leukemia cells [1] and their resistance to therapy [2]. Cytokines secreted by stromal cells, induce homing and keep cells in a quiescent state [3], which in case of chronic myeloid leukemia (CML), remain particularly resistant to most of available chemotherapeutics [4, 5] and are responsible for cancer relapse. Additionally, development of leukemia disturbs the cellular composition of bone marrow [6]. Modification of the extracellular milieu by cytokines secreted by leukemia cells evokes phenotypic changes in surrounding cells that leads to impairment of hematopoiesis [7] and stimulation of angiogenesis [8–12]. Accumulation of highly proliferating cells results in insufficient oxygen and nutrients supplementation and upsets the balance of growth factors. Leukemia cells secrete matrix metalloproteinases (MMPs), which degrade extracellular matrix (ECM) and by this destroy non-cellular components of the niche [13–18] and modify composition of signaling molecules. Altogether it leads to transformation of the bone marrow niche that supports leukemia progression.

Glucose starvation or hypoxia trigger endoplasmic reticulum (ER) stress that activates unfolded protein response (UPR). UPR induces three major stress sensors located within the ER: Ser/Thr protein kinase with endonuclease activity (IRE1), activating transcription factor-6 (ATF6) and protein kinase R-like endoplasmic reticulum kinase (PERK) (reviewed in [19]). One of the main substrates of PERK is Ser 51 of eukaryotic initiation factor 2 α subunit (eIF2α). Phosphorylation of eIF2α inhibits the formation of a ternary complex responsible for initiation of mRNA translation. As a result, translation of most mRNAs is selectively blocked in favor of synthesis of proteins necessary for cell adaptation to stressful conditions. Some proteins are synthesized exclusively upon eIF2α phosphorylation, such as activating transcription factor 4 (ATF4) [20]. The ultimate effect of the UPR activation depends on the severity of the stress stimulus. Upon severe stress, activation of the UPR pathway eventually leads to apoptosis. On the other hand, mild or transient stress results in the adjustment of the cellular protein profile to support cell adaptation and survival.

Induction of mild ER stress was initially observed in cells that form solid tumors. We found that ER stress is also triggered in CML, a non-solid cancer type [21], and evokes sustained activation of UPR and increased phosphorylation of eIF2α (eIF2α-P) in CML cells [22]. Activity of this pathway correlated with leukemia progression and therapy resistance [22, 23] and increase of eIF2α-P in primary Lin-CD34+ cells accompanied CML progression and enhanced their leukemogenic potential [22]. Recent studies confirmed the pro-survival role of eIF2α phosphorylation in leukemia cells upon energetic stress [24]. In parallel, the protective role of eIF2α phosphorylation and ATF4 synthesis in other types of cancers has been recently described [25–28].

Despite the important role that eIF2α-P plays in CML progression, its function in the regulation of the microenvironment of leukemia cells has remained unclear. Here, we show that eIF2α-P affects the composition of proteins secreted by leukemia cells, including enzymes that degrade the extracellular matrix, such as matrix metalloproteinases (MMPs) and cathepsins. We further show that enhanced phosphorylation of eIF2α supports the invasiveness of leukemia cells in a manner dependent on the ATF4 transcription factor. Proteins secreted by CML cells in response to the activation of the eIF2α-P/ATF4 axis increase the invasiveness of human bone marrow stromal fibroblasts. Our findings reveal a key novel function of eIF2α-P in the regulation of the interaction between CML cells and tumor stroma in response to stress and point to ATF4 as a potent therapeutic target.

## RESULTS

### Phosphorylation of eIF2α in K562 cells regulates secretion of extracellular enzymes

**Figure 1:**
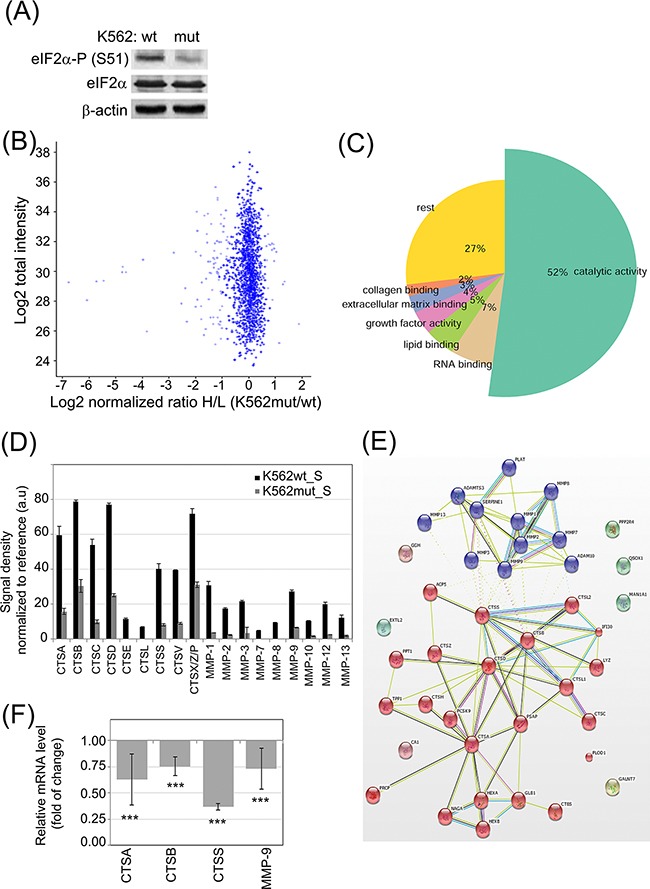
Influence of eIF2α phosphorylation on the profile of enzymes secreted by K562 cells **A.** eIF2α-P level in FACS-sorted K562 expressing GFP alone (K562wt) or GFP and the eIF2α-S51A mutant (K562mut) was analyzed by immunoblot; β-actin is shown as protein loading control. **B-C.** 18h serum-free conditioned medium cleared by multistep centrifugation from K562wt (K562_S) and K562mut (K562mut_S) was analyzed by quantitative SILAC LC-MS/MS. Pooled results from 3 independent experiments are presented. (B) Volcano plot showing normalized ratio of protein abundance as log2 normalized ratio heavy to light (K562mut_S and K562wt_S) (x-axis) and summed peptides intensities as log2 total intensities (y-axis). (C) Gene Ontology assignment of proteins with significantly different abundance between K562wt_S and K562mut_S to biological processes. **D.** Abundance of secreted cathepsins (CTS) and matrix metalloproteases (MMP) were analyzed in S-medium by antibody array. Signal density was analyzed using Image J software and normalized to reference. Graphs are mean protein level ± SEM for n=3 independent samples. **E.** STRING database mapping of proteases identified using SILAC and antibody array. Nodes of clusters are displayed using MCL clustering algorithm with parameter value 2. **F.** mRNA levels of selected enzymes were quantified by real-time RT-PCR and shown as mean fold change ± SEM for n=3 independent samples of K562mut to K562wt cells ***p<0.001 in Student's t-test.

We previously demonstrated correlation between CML progression and increased eIF2α-P [22]. We hypothesized that in addition to its direct effects on CML cells, eIF2α-P might affect the microenvironment of CML through secreted proteins. In order to study the effect of eIF2α-P on the composition of CML secretome, we performed quantitative proteomics on media conditioned by K562 CML cells expressing GFP (K562wt) or a non-phosphorylable mutant of eIF2α (K562mut), which acts as a dominant negative protein and prevents the phosphorylation of endogenous eIF2α [22]. Decrease in eIF2α phosphorylation in total cell lysates of K562mut cells was confirmed by immunoblot (Figure 1A).

In order to quantitatively determine changes in secreted proteins between K562wt and K562mut cells, we used stable isotope labelling by amino acids in cell culture coupled with liquid chromatography-tandem mass spectrometry (SILAC LC-MS/MS) and compared proteins of soluble components from media conditioned by either cell line. To this end, the conditioned media (S) from K562wt (K562wt_S) and K562mut (K562mut_S) cell cultures was cleared of membrane fragments and extracellular vesicles by multistep ultracentrifugation. In total, we identified 2528 proteins using Mascot by at least 2 peptides with a false recovery rate (FDR) of 0.01. Among these, the level of 101 proteins was significantly different between the two conditions. In this group, 83 proteins were decreased and 18 were enriched in media conditioned by K562mut compared to K562wt cells (Figure 1B). Gene Ontology analysis revealed that 52% of proteins, whose concentration was reduced upon decline of eIF2α phosphorylation, possessed catalytic activity (Figure 1C, Table S1) The most abundant enzymes in this group were those involved in the degradation of components of the extracellular matrix (ECM). Apart from ECM-degrading enzymes, there was a significant group of proteins binding to collagen (2%), lipids (5%) and other extracellular matrix components (3%) in a non-catalytic manner. The decrease in the release of enzymes by K562mut cells was further confirmed using antibody arrays (Figure 1D; representative blots are shown in Supplementary Figure S1).

We then used the Search Tool for the Retrieval of Interacting Genes/Proteins (STRING) database to determine if enzymes secreted selectively by K562wt but not K562mut cells interacted physically with one another or were functionally associated. Application of the MCL clustering algorithm yielded two interacting groups – one containing enzymes with MMP activity and another with cathepsin (CTS)-related enzymes (Figure 1E).

We selected CTSs and MMPs for further studies. Apart from MMP-9, the involvement of these proteases in leukemia progression had not been investigated. To determine if the observed changes in CTS and MMP secretion could be attributed to changes at the level of transcription, we measured mRNA levels of selected enzymes by quantitative RT-PCR (Figure 1F). In K562mut cells the level of CTSA, CTSB, CTSS and MMP-9 mRNA was significantly decreased compared to control cells, suggesting that reduced secretion of enzymes is due to decreased transcription of the corresponding genes.

Previously we showed, that in K562 and BV173 cell lines phosphorylation of eIF2α regulates proleukemic pathways at least partially independently on BCR-ABL activity, even upon imatinib treatment [22]. To confirm that secretion of stroma-modifying enzymes can also depend on the phosphorylation status of eIF2α but not solely on the activity of BCR-ABL, the K562wt cells were treated with imatinib, a specific inhibitor of BCR-ABL tyrosine kinase. Whereas BCR-ABL kinase activity was inhibited after 18 hrs incubation with imatinib, as confirmed by the lack of STAT5 phosphorylation, the cells remained alive and the profile of secreted enzymes analyzed by antibody arrays did not change (Supplementary Figure S2A-2C). Therefore we postulate that phosphorylation of eIF2α regulates the secretion of stroma-modifying enzymes in CML cells.

### Decreased eIF2α phosphorylation reduces the invasive potential of leukemia cells

**Figure 2:**
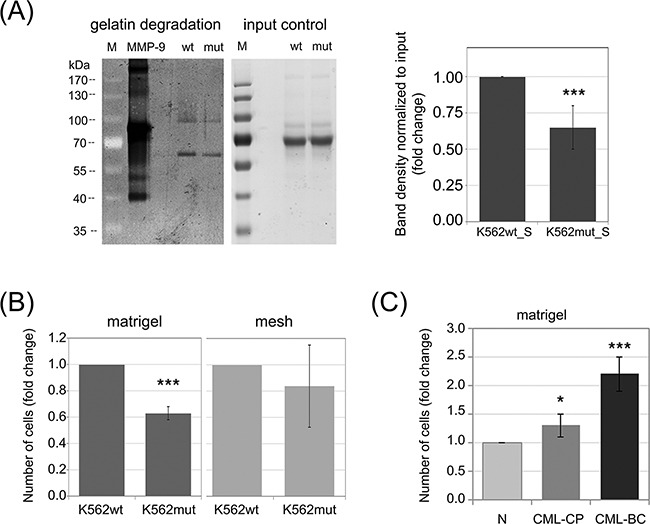
Impact of phosphorylation of eIF2α in leukemia cells on their invasive properties **A.** Gelatinase activity of S-media from K562wt and K562mut cells was analyzed by zymography. Proteins from conditioned media were separated on a polyacrylamide gel containing 5mg/ml gelatin and gelatin digestion was detected by Coomassie staining (dark areas). 5ng of recombinant MMP-9 was used as positive control. Proteins form conditioned media separated on a polyacrylamide gel without gelatin, stained with Coomasie were used as input control; M-molecular weight protein ladder. Graphs show mean fold change of band intensity normalized to input ± SEM, n=3 independent samples. **B.** K562wt and K562mut cell invasion was tested by the transwell assay on matrigel-coated meshes (matrigel, left graph). Migration of cells through the uncoated mesh was measured to account for changes in overall cell motility (mesh, right graph). Graphs represent the mean fold change of the number of cells in the bottom chamber ± SEM, n=3. **C.** Invasion of Lin-CD34+ normal (N) and CML primary cells from chronic phase (CML-CP) and blast crisis (CML-BC) was measured as in B. ***p<0.001, *p<0.05 in Student's t-test.

Differences in the abundance of secreted proteases could lead to changes in the ability of CML cells to degrade the ECM. One of the main components of the ECM is collagen and its derivative - gelatin. Both CTSs and MMPs, the two groups of secreted enzymes identified in our proteomic screen, possess collagenolytic and gelatinolytic activity. We therefore hypothesized that conditioned media from K562mut cells would have reduced ability to digest gelatin. We verified this hypothesis using gelatinase zymography. We found that the efficiency of gelatin degradation by K562mut_S was significantly decreased compared to K562wt_S (Figure 2A). There were two bands visible in the gelatin-polyacrylamide gel, suggesting that the samples contained gelatinases with molecular weights of around 90kDa and 60kDa, respectively. The 90kDa gelatinase could represent MMP-9, and the ~60kDa band could correspond to one of several MMPs: MMP-2, MMP-8 or MMP-14. Of these latter three, however, only MMP-2 has gelatinolytic activity, suggesting that it is likely the 60kDa gelatinase.

Since K562mut cells degrade the ECM at a slower rate than K562wt, we further hypothesized that the reduced eIF2α-P in K562mut cells may result in the impairment of their invasive potential. To test this hypothesis, we used the BD Biocoat Tumor Invasion System. This system measures the ability of cells to penetrate from the upper chamber to the lower chamber of a transwell through a mesh coated with matrigel. An uncoated mesh is used as control to distinguish between bona fide invasion and simple migration. The number of cells that invaded into the bottom chamber through matrigel coating after 18h was significantly lower for K562mut cells than for K562wt cells (Figure 2B, matrigel). On the other hand, the number of cells that migrated through the uncoated mesh was not significantly different between the two cell lines (Figure 2B, mesh). This suggests that reduced phosphorylation of eIF2α may impair the invasiveness of leukemia cells.

We previously showed that progression of CML from chronic phase to blast crisis is accompanied by increased eIF2α phosphorylation [22]. Moreover, CML progression is known to be associated with increased invasiveness of the cancer cells [14]. We therefore expected that leukemia cells at blast crisis would show higher invasiveness in the transwell assay than chronic phase cells or untransformed healthy cells. We verified this hypothesis using Lin-CD34+ primary cells from healthy donors or CML patients. The cells at blast crisis (CML-BC) showed over 2-fold higher invasion potential than normal cells from healthy donor (N) and 1.85-fold higher than cells at chronic phase (CML-CP) (Figure 2C). Taken together, this data suggests that increased phosphorylation of eIF2α in leukemia cells could support invasiveness of cancer cells and in that way play a role in CML progression.

### eIF2α-P-dependent secretome of CML cells evokes ECM degradation by fibroblasts and their invasive potential

Stimulation of phenotypic changes of bone marrow fibroblasts was demonstrated to accompany progression of multiple types of leukemia, such as multiple myeloma [29] and chronic lymphocytic leukemia [30]. In order to study the influence of soluble factors secreted by K562 cells on stromal cells, we added conditioned media from K562wt and K562mut cultures to cultures of HS-5 cells, a line of immortalized human bone marrow stromal fibroblasts. The HS-5 line can support the growth of hematopoietic progenitor cells [31] and is commonly used as a model of bone marrow stroma. Conditioned media (S) from HS-5 (HS-5_S), K562wt (K562wt_S) and K562mut (K562mut_S) cell cultures was cleared by multistep ultracentrifugation and used for further assays.

**Figure 3:**
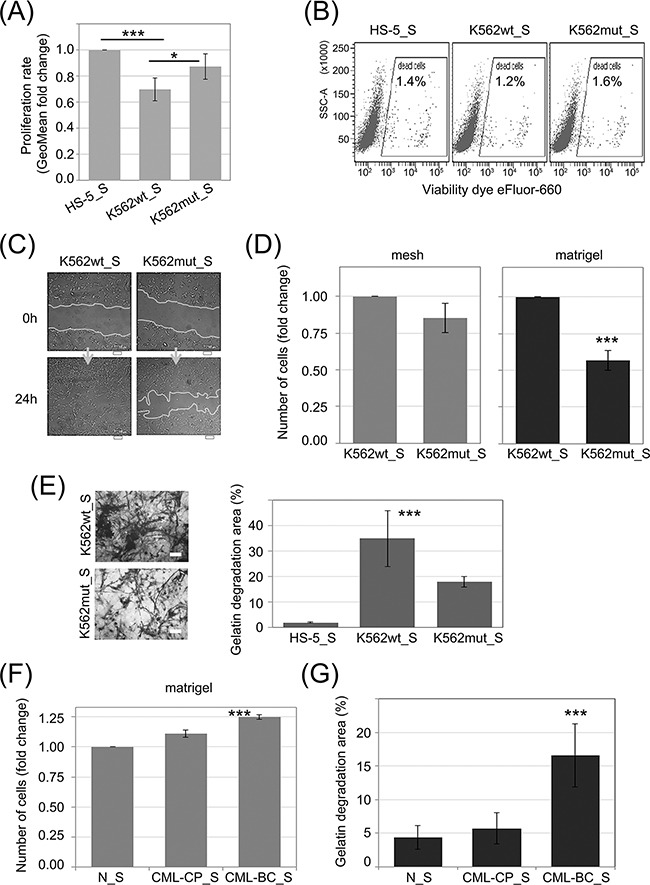
Influence of eIF2α phosphorylation on the invasion potential of HS-5 stromal fibroblasts HS-5 cells were cultured in conditioned media cleared by multistep centrifugation (K562wt_S, K562mut_S or HS-5_S). **A.** Proliferation rate was assayed by flow cytometry. Bars correspond to mean fold change of proliferation dye fluorescence (GeoMean) at 24h and 72h ± SEM; n=4 **B.** Flow cytometry analysis of cell death levels in HS-5 cells cultured in the presence of conditioned media from different cultures. Typical scatterplots for each culture condition are shown, with side scatter area (SSC-A; y-axis) plotted against viability dye eFluor 660 signal intensity. Gate used to detect dead cells is indicated on each plot **C.** Monolayers of HS-5 cells cultured in the presence of K562wt_S or K562mut_S were subjected to the wound healing assay. Representative images out of 3 independent experiments of wound closure immediately after wounding (0h) and after 24h are shown. Scale bar = 100 μm **D.** HS-5 cells were subjected to the transwell invasion assay as in Figure 2B, n=3 **E.** Gelatin degradation by HS-5 cells cultured in S medium, as indicated, was quantified using OregonGreen-labeled gelatin. Representative images of fluorescent gelatin (grey) are shown in the left panel. Gelatin degradation is apparent as black areas on a grey background. Scale bar = 10 μm. Right panel shows mean percentage of gelatin degradation area normalized to cell number ± SEM; n=3 independent experiments **F, G.** Matrigel invasion (F) and gelatin degradation (G) assays were performed as in D. and E., respectively, on HS-5 cells cultured in media conditioned by Lin-CD34+ cells from a healthy donor (N_S), chronic phase CML (CML-CP_S) or blast crisis (CML-BC_S). ***p<0.001 in Student's t-test.

We first tested the ability of CML-conditioned media to regulate proliferation (Figure 3A) and cell death (Figure 3B) of stromal fibroblasts. K562wt_S, but not K562mut_S, significantly reduced the rate of proliferation of HS-5 cells (Figure 3A). In contrast, cell death rates remained constant under all conditions as indicated on the dot plots (Figure 3B). We then verified if components secreted by K562 cells could affect fibroblast migration and invasiveness. K562wt_S-exposed HS-5 cells had increased velocity of wound closure compared to HS-5 cells exposed to K562mut_S (Figure 3C). Next we found that migration rate of HS-5 cells was not significantly changed in the presence of soluble factors secreted by K562wt compared to K562mut cells (Figure 3D, mesh). However, K562wt_S significantly increased the ability of HS-5 cells to penetrate through matrigel in a transwell assay (Figure 3D, matrigel). Next, to confirm that factors secreted by K562 cells have an impact on ECM degradation by HS-5 cells, we measured the ability of cells cultured in the presence of different conditioned media to degrade fluorescent gelatin (Figure 3E). We found that K562wt_S and K562mut_S enhanced the ability of HS-5 cells to degrade gelatin, but the effect evoked by K562wt_S was significantly (~2-fold) higher than that evoked by K562mut_S (Figure 3E).

We then wanted to see if the effects on HS-5 cell invasiveness caused by K562 cell conditioned media can be recapitulated by media collected from primary leukemia cell cultures (Figure 3F and 3G). Indeed, media conditioned by blast crisis leukemia Lin-CD34+ cells increased the ability of HS-5 cells to both penetrate through matrigel in a transwell assay (Figure 3F) and to degrade fluorescent gelatin (Figure 3G) compared to media conditioned by Lin-CD34+ cells from chronic phase or a healthy donor. In summary, these results suggest that modification of protease secretion by eIF2α phosphorylation in leukemia cells, which accompanies disease progression, may affect the invasive potential of not only leukemia cells themselves, but also of surrounding cells in the tumor stroma.

### eIF2α phosphorylation-dependent ATF4-mediated regulation of enzyme secretion by leukemia cells

**Figure 4:**
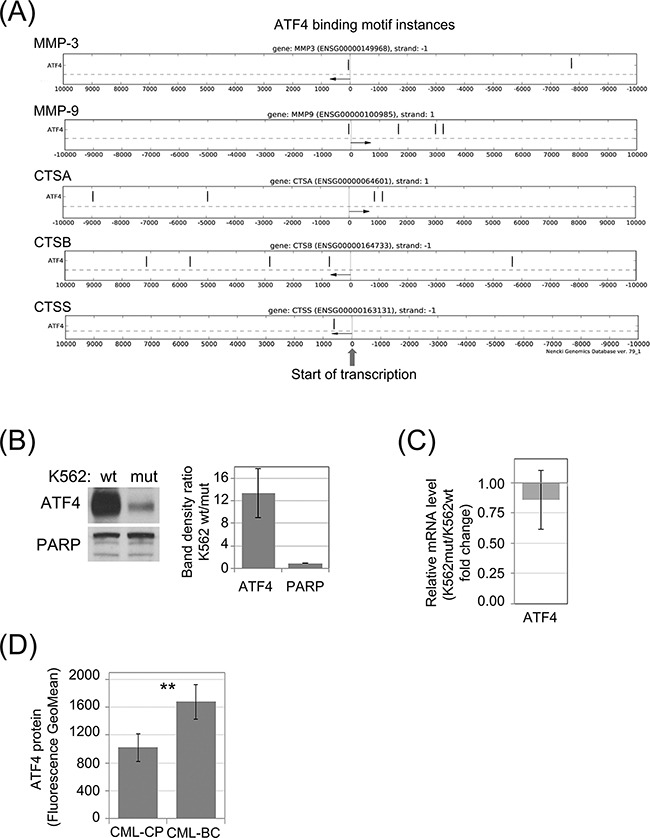
Synthesis of ATF4 protein in leukemia cells is dependent of eIF2α phosphorylation **A.** Genomic locations of potential ATF4 binding motifs (black vertical dashes) in experimentally verified gene regulatory regions within 10kb from transcription start sites (TSS) of selected protease genes. Distance from the TSS is indicated on the X-axis. Direction of transcription is marked with an arrow. **B.** Protein level of ATF4 in K562wt and K562mut cells analyzed by immunoblot; PARP was used as loading control. Band density was quantified and expressed as mean ratio in K562wt to K562mut cells ± SEM; n=3 **C.** Level of ATF4 mRNA was determined by real-time RT-PCR, and is shown as fold change of K562mut to K562wt cells. **D.** Level of ATF4 protein in Lin-CD34+ primary cells from CML-CP and CML-BC was analyzed by flow cytometry. Fluorescence GeoMean values of CML-BC to CML-CP ± SEM; n=3 independent samples. **p<0.005 in Student's t-test.

Phosphorylation of eIF2α was demonstrated to modify level of ATF4 protein synthesis at the step of mRNA translation [32]. On the other hand, according to the Nencki Genomics Database [33], which contains consensus sequence of regulatory regions (Supplementary Figure S3), there are motifs possibly recognized by ATF4 transcription factor in selected CTSs and MMPs. The schematic representation of the likely functional ATF4 binding motif instances in the experimentally identified gene regulatory regions: TF bindings sites and DNase1 open chromatin regions, FANTOM promoters and enhancers are presented in the Figure 4A. This analysis suggests that decreased level of ATF4 in cells with reduced eIF2α phosphorylation could account for the changes in protease secretion (Figure 1D) and mRNA levels (Figure 1F). Indeed, we observed reduction of ATF4 protein in K562mut cell lysates by immunoblot (Figure 4B). In agreement with previous reports [32] this effect did not result from reduced ATF4 mRNA level (Figure 4C). Based on these results, we hypothesized that ATF4 levels would increase as a function of CML progression. Indeed, primary blast crisis CML cells showed 1.45-fold higher levels of ATF4 than chronic phase CML cells in a flow cytometry assay, used instead of Western blotting due to limited number of cells (Figure 4D).

**Figure 5:**
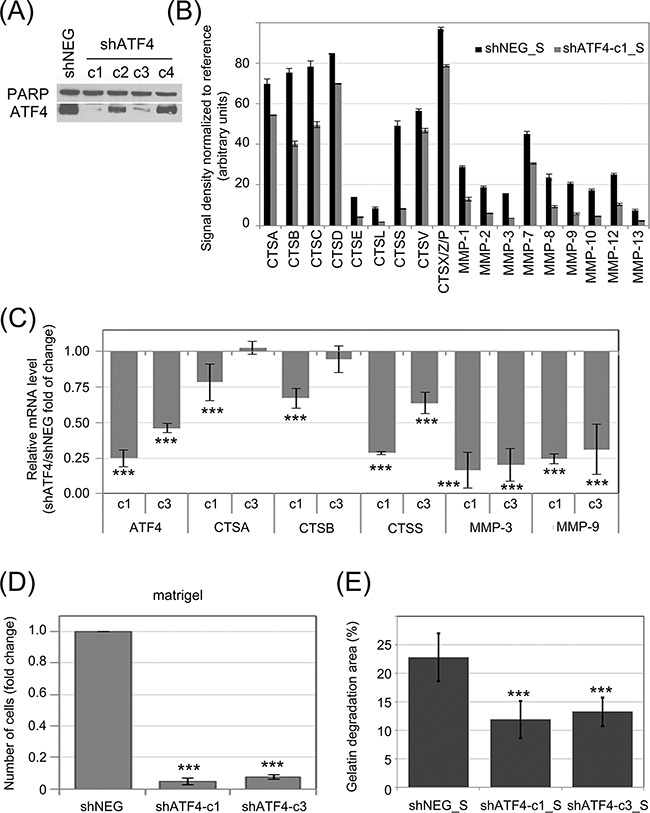
Influence of ATF4 on protease secretion and invasive potential of CML cells and stromal fibroblasts ATF4 was depleted in K562 cells by viral transduction of 4 different clones of shRNA specific to ATF4 (shATF4-c1 – c4); Non-targeting shRNA (shNEG) was used as control **A.** ATF4 protein level was quantified in whole cell lysates by immunoblot; PARP was used as loading control. **B.** Protease abundance was measured by antibody array in serum-free conditioned media from cultures of K562 expressing shATF4 (shATF4-c1_S) and control shRNA (shNEG_S). Signal density was analyzed using Image J software and normalized to reference. Graphs are mean protein level ± SEM for n=3 independent samples **C.** Level of mRNA of selected enzymes was measured by real-time RT-PCR and is shown as mean fold change between K562 cells expressing shATF4-c1 or shATF4-c3 and shNEG ± SEM for n=3 independent samples **D.** Matrigel invasion of K562 expressing the indicated shRNA constructs was measured using the transwell assay, as in Fig. 2B; n=3 independent experiments **E.** Gelatin degradation by HS-5 cells cultured in shATF4-c1_S or shATF4-c3_S and shNEG_S was measured as in Fig. 3E; n=3. ***p<0.001, *p<0.05 in Student's t-test.

In order to investigate the influence of ATF4 on protease secretion, K562 cells were transduced with shRNA specific to ATF4 mRNA (shATF4) to downregulate its expression, or a negative control shRNA (shNEG). The efficiency of ATF4 knockdown by different shRNAs was deteced by real time RT-PCR (Figure 5C) and confirmed by immunoblot (Figure 5A). This showed that two shRNAs (named as c1 and c3) were sufficiently silencing ATF4, among which shRNA-c1 was the most effective at both, protein (Figure 5A) and mRNA (Figure 5C) levels. We then measured the effect of ATF4 silencing on CTS and MMP secretion by antibody array. The secretion of proteases by K562 cells was significantly reduced in cells expressing ATF4 shRNA-c1 (Figure 5B; representative blots are presented in Supplementary Figure S4). Consistently, mRNA levels of all of the tested protease genes were also significantly downregulated in cells with ATF4 knockdown mediated by shRNA-c1 (Figure 5C), suggesting that ATF4 may regulate the expression of proteases at the level of transcription. As the ATF4 silencing mediated by the shRNA-c3 was lower compared to shRNA-c1, the effect on the enzymes expression was moderated in case of CTSA and CTSB, however still significant in case of CTSS, MMP9 and MMP3 enzymes.

To determine the role of ATF4 in proliferation and sensitivity to imatinib, the shNEG and shATF4 transduced K562 cells (c1 and c3) were untreated and treated with different concentrations of imatinib. In cells depleted with ATF4, the apoptosis rate in the AnxA5 assay was not increased as well as the clonogenic potential in the presence and absence of imatinib treatment was not reduced (Supplementary Figure S5A and S5B). Therefore, ATF4 does not regulate the proliferation of CML cells and their sensitivity to imatinib.

To verify the influence of ATF4 activity on the invasive potential of leukemia cells, we performed the matrigel transwell assay (Figure 5D). Cells expressing ATF4 shRNA, both, c1 and c3, had strongly reduced ability to invade through matrigel compared to control cells. We also hypothesized that media conditioned by K562 cells with ATF4 knockdown may have reduced ability to promote fibroblast invasiveness. Indeed, HS-5 cells cultured in media conditioned by K562 cells with ATF4 knockdown (shATF4_S), clones c1 as well as c3, degraded fluorescently labeled gelatin at a significantly slower rate than those cultured in media conditioned by control K562 cells (Figure 5E). Altogether, our results show that the effect of eIF2α phosphorylation on protease secretion is ATF4-dependent. The eIF2α-P/ATF4 axis is likely to play a central role in the potentiation of invasive properties of leukemia cells, as well as influence their cross-talk with bone marrow stromal fibroblasts.

## DISCUSSION

The interactions between cancer cells and their microenvironment are crucial for the development of leukemia. A variety of cytokines, growth factors, adhesion molecules and ECM remodelling proteins are secreted by both tumor and non-tumor cells, mediating cellular communication within the cancer microenvironment and providing a suitable niche for cancer cell persistence. This crosstalk is also involved in disease progression and development of resistance to therapy.

We previously showed that enhanced phosphorylation of eIF2α in CML cells potentiates leukemogenesis and accompanies CML progression from chronic phase to blast crisis [22]. Here, we provide evidence suggesting that induction of stress response in CML cells, which is associated with eIF2α phosphorylation, participates in the remodeling of extracellular milieu, which can contribute to cancer development. We demonstrate that reduced phosphorylation of eIF2α in CML cells correlates with decreased secretion of ECM-modifying enzymes and blocks cells invasiveness. This is in agreement with a recent report showing that triggering of ER stress and activation of the PERK/eIF2α-P axis is essential to the epithelial-to-mesenchymal transition [34], a process that promotes invasiveness. Importantly, inhibition of BCR-ABL activity by imatinib was not able to diminish amounts of secreted enzymes confirming that eIF2α–dependent signaling is responsible for such regulation.

Moreover, we demonstrate for the first time the induction of the invasive potential of bone marrow stromal fibroblasts upon factors secreted by CML cells in an eIF2α-P dependent manner. This phenomenon may assist the disruption of bone marrow architecture during leukemia progression and facilitate liberation of cancer cells to the bloodstream. It was shown recently that secretion of cytokine-like leukemia inhibitory factor 1 evokes invasiveness of stromal fibroblasts by turning them into the cancer associated fibroblasts (CAFs) [35]. Induction of phenotypic changes in bone marrow fibroblasts was demonstrated to promote progression of multiple myeloma [29] and chronic lymphocytic leukemia [30, 36]. CAFs were also shown to assist the invasion of cancer cells in breast cancer [37] or gastric carcinoma [38].

We show that phosphorylation of eIF2α in CML cells modifies the expression and secretion of proteases belonging to the cathepsin and matrix metalloprotease families. Moreover, the increased activity of these enzymes supports extracellular matrix degradation by fibroblasts. These findings are in agreement with the previous observations that stimulation of secretion of MMP-9 accompanies CML progression and mediates the degradation of extracellular matrix [13–15]. Increased secretion of MMP-9 by chronic B-lymphocytic leukemia (B-CLL) was also shown to play a significant role in the invasiveness of these cells [16]. Other MMPs that we also showed to be regulated by eIF2α-P, MMP-7 [17] and MMP-12 [18], were found to potentiate invasion of leukemia cells. MMP-3, also known as Stromelysin-1, another protease identified in our screen, has the ability to cleave a broad spectrum of ECM components [39] and activates other MMPs such as MMP-9 [40] and MMP-7 [41]. Simultaneous up-regulation of MMPs was observed in several diseases [42–44]. Contrary to MMPs, the significance of cathepsins released by leukemia cells has not been studied so far. Nevertheless, increased level of secreted CTSS or CTSB have been shown to be associated with metastasis in other cancer types [45–47]. Furthermore, CTSX is upregulated in metastatic gastric cancer [48], enhanced levels of CTSB and CTSL accompany malignant ovarian tumors [49], and CTSS directly supports invasion of hepatocellular carcinoma cell lines [50]. It is worth noting that cathepsins display the highest activity in the acidic and low oxygen conditions, which occur in dense solid tumors but also in the bone marrow niche. Taking together, cathepsins secreted by leukemia cells could play a significant role in supporting the invasiveness of CML cells, which might be especially important under the hypoxic state in the bone marrow.

Finally, we demonstrated that the effects of eIF2α phosphorylation on protease secretion, but not on imatinib sensitivity are likely mediated by ATF4. We postulate that ATF4 production in leukemia cells plays a significant role in the regulation of extracellular matrix dynamics and invasiveness of both CML and stromal cells. Our claims are supported by the recently reported involvement of ATF4 in metastasis of different tumors. First, circulating metastatic cancer cells have increased level of ATF4 [51]. Moreover, hypoxia occurring in breast cancer stimulates the migration of cancer cells upon activation of the PERK/eIF2α-P/ATF4 signaling pathway [52]. Furthermore, up-regulation of ATF4 in osteosarcoma cells accompanies tumor progression and supports metastasis [53]. In addition, ATF4 was postulated to stimulate metastasis in human fibrosarcoma cells [54]. Finally, increased expression of ATF4 in esophageal squamous cell carcinoma correlates with invasion and metastasis, an effect attributed to increased transcription of MMP-2 and MMP-7 [55]. This is consistent with our findings that MMP-2 and MMP-7 are downregulated upon ATF4 knock-down. All these data strongly support the pro-invasive role of ATF4, and are in full agreement with our results.

Our current and previous data identified the PERK-eIF2α-ATF4 axis as an interesting target for pharmacological intervention in CML. PERK inhibitors, GSK2606414 and its derivative GSK2656157 optimized for clinical investigation have been tested in different types of tumors such as pancreatic cancer and multiple myeloma [56, 57]. These compounds showed high therapeutic potential, however their still require safety and toxicity evaluation.

## SUMMARY

We demonstrate that the enhanced phosphorylation of eIF2α is associated with secretion of enzymes that catalyze extracellular matrix degradation, thus inducing cancer-supportive changes in the microenvironment. Phosphorylation of eIF2α in CML cells potentiates the invasiveness of leukemia cells as well as of bone marrow stromal fibroblasts. Modification of ATF4 protein levels in an eIF2α-dependent manner is likely to account for the observed effects on protease expression and secretion. Overall, our results point to eIF2α and ATF4 as potential therapeutic targets in leukemia.

## MATERIALS AND METHODS

### Cell culture and treatment

K562 cells (ATCC®CCL-243) and HS-5 cells (ATCC®CRL-11346) were obtained from American Type Culture Collection. K562 and HS-5 cell lines were cultured in RPMI-1640 (SIGMA) supplemented with 100U/ml penicillin, 100μg/ml streptomycin, 2mM L-glutamine (SIGMA) and 10% FBS (v/v, Biowest). CML-CP cells from patients at diagnosis (n=3) and CML-BC cell samples (n=3) were obtained from Stem Cell and Leukemia Core Facility of the University of Pennsylvania (Philadelphia, PA), and normal hematopoietic cells from healthy donors were purchased from Cambrex Bio Science (Walkersville, MD). Lin-CD34+ cells were selected by magnetic sorting using the EasySep negative selection human progenitor cell enrichment cocktail followed by human CD34 positive selection cocktail (#19056 and #18056, respectively; StemCell Technologies). Lin-CD34+ cells were cultured in StemSpan H3000 medium (StemCell) supplemented with 1ng/ml SCF, 0.2ng/ml IL-3, 0.2ng/ml IL-6, 1ng/ml FlT3 (PeproTech). Cells were grown under standard conditions 37°C, 5% CO_2_.

### Transfection

Viral vector pMSCV-IRES-GFP or pMSCV-IRES-GFP/eIF2α-S51A that express both GFP and untagged eIF2α-S51A were expressed in K562 cells as described earlier [58]. Cell expressing only GFP are denoted in this manuscript as K562wt and cells expressing eIF2α-S51A and GFP are described as K562mut. GFP-expressing cells were sorted using FACS Aria (BD).

### Creation of stable ATF4 knock-down K562 cell line

ATF4 was silenced in human K562 cells with MISSION TRC shRNA Lentiviral Particles (#SHCLNVNM_001675/TRCN0000013573, SIGMA); 5 different clones. For control infection, MISSION TRC2 pLKO.5-puro Non-Mammalian shRNA Control Transduction Particles were used (#SHC202V, SIGMA). Cells were seeded in 96-well plate (8000 cells/well) and transduced with lentiviral particles at multiplicity of infection of 5. After 3 days, puromycin was added at 1μg/ml. Antibiotic selection was performed for 10 days. As infection with shATF4-c5 turned to be lethal, only shATF4-c1-c4 were used for experiments.

### Conditioned medium collection

For conditioning, cells were suspended in serum-free medium (unless otherwise stated) at a density of 4×10^5^ cells/mL. After 18h, conditioned medium was collected and cleared from cellular debris and extracellular vesicles during multistep centrifugation at 4°C as follows: (1) 163xg for 5min; (2) 319xg for 5min; (3) 1277xg for 20min; (4) 10000xg for 40min; (5) 110000xg for 1h 40min (45Ti rotor, Beckman Coulter). Supernatant from the last centrifugation is designated as “S” throughout the manuscript and was used for experiments.

### SILAC LC-MS/MS

Composition of secreted proteins in conditioned medium was studied after stable isotope labelling by amino acids in cell culture (SILAC) as described by Ong et al. [59]. Briefly, the cells were cultured in SILAC DMEM without L-Arginine and L-Lysine (Thermo Scientific) with 10% dialyzed FBS (Thermo Scientific) ‘heavy medium’ supplemented with stable isotope labelled ^13^C_6_ L-Arginine at 0.47mM and ^13^C_6_^15^N_2_ L-Lysine at 0.46mM final concentration (#CLM-2265-H-0.1 and #CNLM-291-H-0.1 accordingly from Cambrige Isotope Laboratories Inc.) or ‘light medium’ with non-labelled L-Lys and L-Arg (SIGMA) added at the same final concentration. After 6 population doublings (FACS analysis showed about 24h per 1 doubling) labelled cells were collected, washed in serum-free SILAC DMEM and suspended in serum free medium heavy for K562mut or light for K562wt for 18h (cells density 6×10^5^/ml). The experiment was repeated twice performing SILAC also in reverse mode. Efficiency of proteins labelling was verified to be above 98%.

Soluble proteins from S from each variant were mixed in 1:1 ratio normalized to the number of cells. Samples were processed using filter-aided sample preparation (FASP) according to [60], and subjected to in-gel isoelectric focusing of peptides as described [61]. Each gel strip was then cut into 21 fragments. Peptides eluted from each fragment were separately subjected to separation by high pressure liquid chromatography (HPLC) and mass spectrometry analysis using LTQ Orbitrap Velos (Thermo Fisher Scientific). Data analysis was done using Mascot software followed by MaxQuant software for quantitative data analysis as previously described [62].

### Antibody arrays

The profile of secreted proteases in serum-free S was determined using Human Protease Array Kit (#ARY021, R&D Systems) according to the manufacturer's protocol. Densitometry was performed using ImageJ software (National Institutes of Health) with Protein array analyzer plug-in. The values were normalized to density of 6 reference spots included in each membrane. The list of differentially secreted proteins identified by SILAC LC-MS/MS and antibody arrays was subjected to physical and functional protein associations network search using STRING (http://string-db.org).

### Wound-healing assay

Migration was assayed by wound healing experiments as previously described [63]. Time-lapse observations were performed for 48h using Leica AF7000 Live Imaging System (Leica Microsystems) microscope equipped with an environmental chamber, and a 10x/0.40NA objective. Wound closure was calculated and expressed as percentage of the area of the initial wound (defined at 0 time point). Image analysis was performed using ImageJ.

### Trans-well migration and invasion assay

Cell migration and invasion were examined using inserts with polycarbonate filters with FluoroBlock membrane (8μm pore size; BD) either uncoated or coated with Matrigel (BD) following the manufacturer's protocol. Cells were labelled with long term cell tracker Dil (Life Technology) and seeded into the upper chamber in 24h serum-free conditioned medium S. The lower chamber was filled with 10% FBS 24h S. After 20h of incubation, the invasive cells on the underside were visualized and counted under the Leica fluorescent microscope DMI6000 using a 10x objective (Leica Microsystems) in 16 random fields. The number of cells was quantified with ImageJ.

### Fluorescent gelatin degradation assay

The matrix degradation assay was conducted as described previously [61], with modifications. Briefly, 1.5×10^4^ cells stained with cell tracking dye CMAC (Life Technology) were seeded in serum-free conditioned S medium on dishes with glass-bottom (MatTek Corporation) coated with gelatin labelled with OregonGreen (Life Technologies) according to the manufacturer's protocol. Then cells were cultured for 18h and images were taken with a Leica fluorescent microscope with a 10x objective. By using the Image J Analyze Particles plugin the total area covered by degradation holes in the fluorescent background was measured in multiple fields and normalized to the total number of seeded cells.

### Zymography

Equal amount of protein from each serum free S medium (1.5mg) was subjected to gelatinase enrichment step using gelatin sepharose beads (GE Healthcare) for 1.5h with rotation at 4°C. Then the beads were washed and gelatinases were eluted for 45 min with 10% DMSO in WB buffer (50mM Tris-HCl pH 7.5, 150mM NaCl, 5mM CaCl2, 0.02% NaN3) with rotation at 4°C, followed by centrifugation at 12000xg for 15min at 4°C. The supernatant was mixed with loading buffer (62.5mM Tris-HCl pH 6.8, 2% SDS, 10% glycerol, 0.01% bromophenol blue) and loaded on 8% polyacrylamide gel containing 5mg/ml gelatin (Sigma Aldrich). As positive control, 5ng of recombinant MMP-9 was used. Following electrophoresis at 100V, the gel was washed twice in 2.5% Triton-X100 for 25min at room temperature followed by 60h incubation at 37°C in the activity buffer (50mM Tris-HCl pH 7.5, 10mM CaCl2, 1% Triton-X100, 0.02% NaN3) with gentle agitation. The areas of gelatin degradation area were visualized using Coomasie Brilliant Blue staining. In parallel, the input control samples (collectec before addition of gelatin beads) were heat denatured and subjected to SDS-PAGE in 8% gel, followed by staining with Coomasie Brilliant Blue. Gel images were scanned using CanoScan Lide210 (Canon) and bands density was analyzed using Image J.

### Western blotting

Cells were washed with phosphate-buffered saline (PBS), pH 7.4 prior to lysis at 95°C in loading buffer containing: 50mM Tris-HCl pH 6.8, 10% glycerol and 2% SDS. After SDS-PAGE and transfer onto nitrocellulose, proteins were immunostained with the primary antibodies: mouse anti-β-actin (SIGMA), rabbit anti-phospho-eIF2α (S51) (Invitrogen), mouse anti-eIF2α (Cell Signaling), mouse anti-GAPDH (Merck Millipore), rabbit anti-ATF4 (ProteinTech), rabbit anti-PARP (Cell Signaling); followed by secondary antibodies conjugated with horseradish peroxidase (GE Healthcare). The proteins were visualized using ECL kit according to the manufacturer's instructions.

### RNA isolation and real-time RT-PCR

Total RNA was isolated using TRIzol Reagent (SIGMA). 1μg of RNA was subjected to reverse transcription with M-MLV enzyme (Promega) and oligo-dT primers (SIGMA). Real-time PCR was performed using SensiFAST SYBR Kit (Bioline) on the 7900HT Fast Real-time PCR System (Applied Biosystems). Primers used for real-time PCR are presented in Supplement Table S2. After normalization to 18SrRNA the comparative 2^−DD^Ct method was used to determine relative mRNA level [64].

### FACS analysis of ATF4 protein level

The cells were fixed and permeabilized using PerFix EXPOSE kit from Beckman Coulter (#PN B26976), according to the manufacturer's protocol. The cells were stained for 30 min at room temperature with the primary antibody against ATF4 conjugated with fluorochrome - Allophycocyanin using Zenon labelling kit (Life Technologies) appropriate for the antibody isotype. Isotype control antibody (Abcam) was labelled and used as a background staining control. Flow cytometry analysis was performed using LSR Fortessa (BD).

### Cell death assay

Cell were labeled with viability dye eFluor 660 (eBioscience), according to the manufacture's protocol, followed by analysis of cell death by flow cytometry (LSR Fortessa).

### Proliferation assay

Cells were labelled with 10μM cell proliferation dye eFluor 670 (eBioscience) according to the manufacture's protocol and then cultured for 72 hours in conditioned medium. Fluorescence intensity of the cells was measured by flow cytometry (LSR Fortessa) on the staining day and in 24 h intervals.

### Statistics

All data are shown as mean ± SEM of at least three independent experiments (with duplicates included in each experiment). Student's t-test or one-way analysis of variance (ANOVA) were used to verify statistical significance; *p<0.05; **p<0.005; ***p<0.001.

## ETHICAL APPROVAL STATEMENT

Studies were approved by the Temple University Institutional Review Board (#11492).

## SUPPLEMENTARY FIGURES AND TABLES




