# Supplementary material for: Increased phosphorylation of eIF2α in chronic myeloid leukemia cells stimulates secretion of matrix modifying enzymes
Source: Oncotarget. 2016 Oct 27;7(48):79706–21. doi: 10.18632/oncotarget.12941 (PMC5346746; doi:10.18632/oncotarget.12941)
Supplement: Supplementary file 1 [file oncotarget-07-79706-s001.pdf]

Increased phosphorylation of eIF2α in chronic myeloid leukemia cells stimulates secretion of matrix modifying enzymes

SUPPLEMENTARY FIGURES AND TABLES

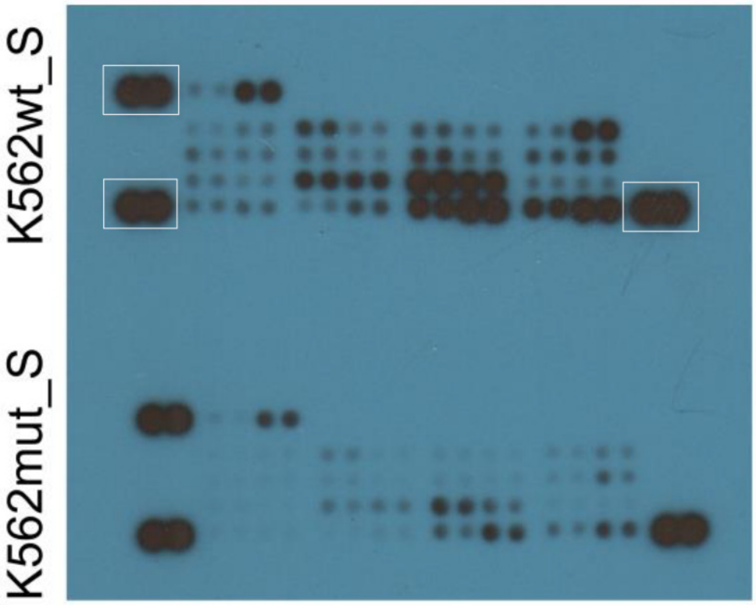

Supplementary Figure S1: Human Protease Array Scan pictures of membranes incubated in conditioned medium by K562wt or K562mut cells (K562wt_S and K562mut_S, respectively). Membrane reference dots included in the membrane are marked with white square.

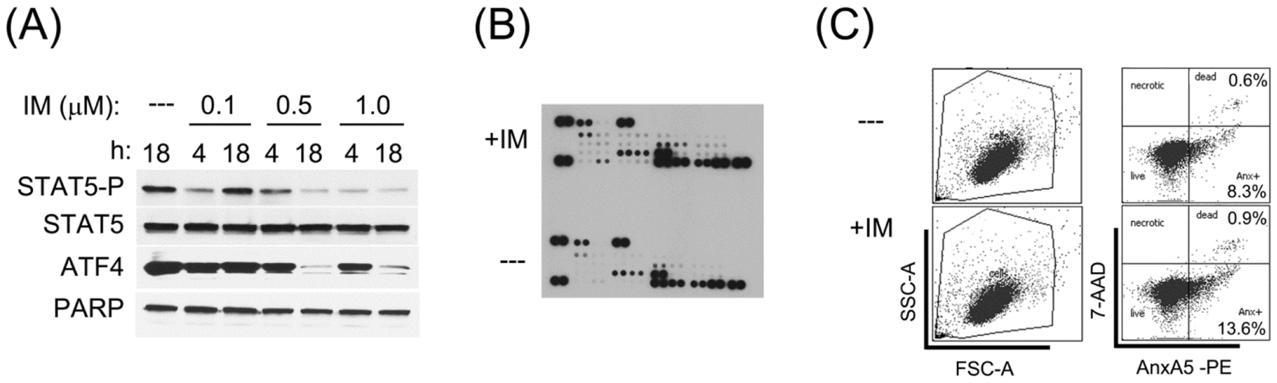

Supplementary Figure S2: Influence of imatinib on proteases secretion by K562wt cells. A. Protein level of STAT5 total (antibody #9363 Cell Signaling) and phosphorylated at Y694 (antibody #9356, Cell Signaling) in untreated (---) or imatinib (IM) treated K562wt cells analyzed by Western blotting in whole cell lysates. Imatinib given at final concentrations: 0.1, 0.5 or 1.0 μM for 4 or 18h. PARP was used as loading control. **B.** Protease abundance was measured by antibody array in the serum-free conditioned media from cultures of K562wt control (---) and treated with 0.5 μM imatinib for 18h (+IM). Representative immunoblots are presented. **C.** Induction of apoptosis in K562wt cells cultured in serum-free medium for 18h untreated or treated with 0.5 μM imatinib was verified by AnxA5-PE/7-AAD staining and FACS analysis and compared to untreated cells. Dot plots in the left panel show population distribution according to forward and side scatter (SSC to FSC). In the right panel presented is staining with 7-AAD (y-axis) and AnxA5-PE (x-axis) to distinguish between four populations of cells: live (unstained cells in left lower quadrant); necrotic (only 7-AAD positive cells in left upper quadrant); early apoptotic (only AnxA5-PE positive cells in right lower quadrant); late apoptotic, dead (7-AAD and AnxA5-PE positive cells in right upper quadrant). Number of AnxA5-PE positive cells expressed as % of total cell population analyzed is shown in the corresponding gates.

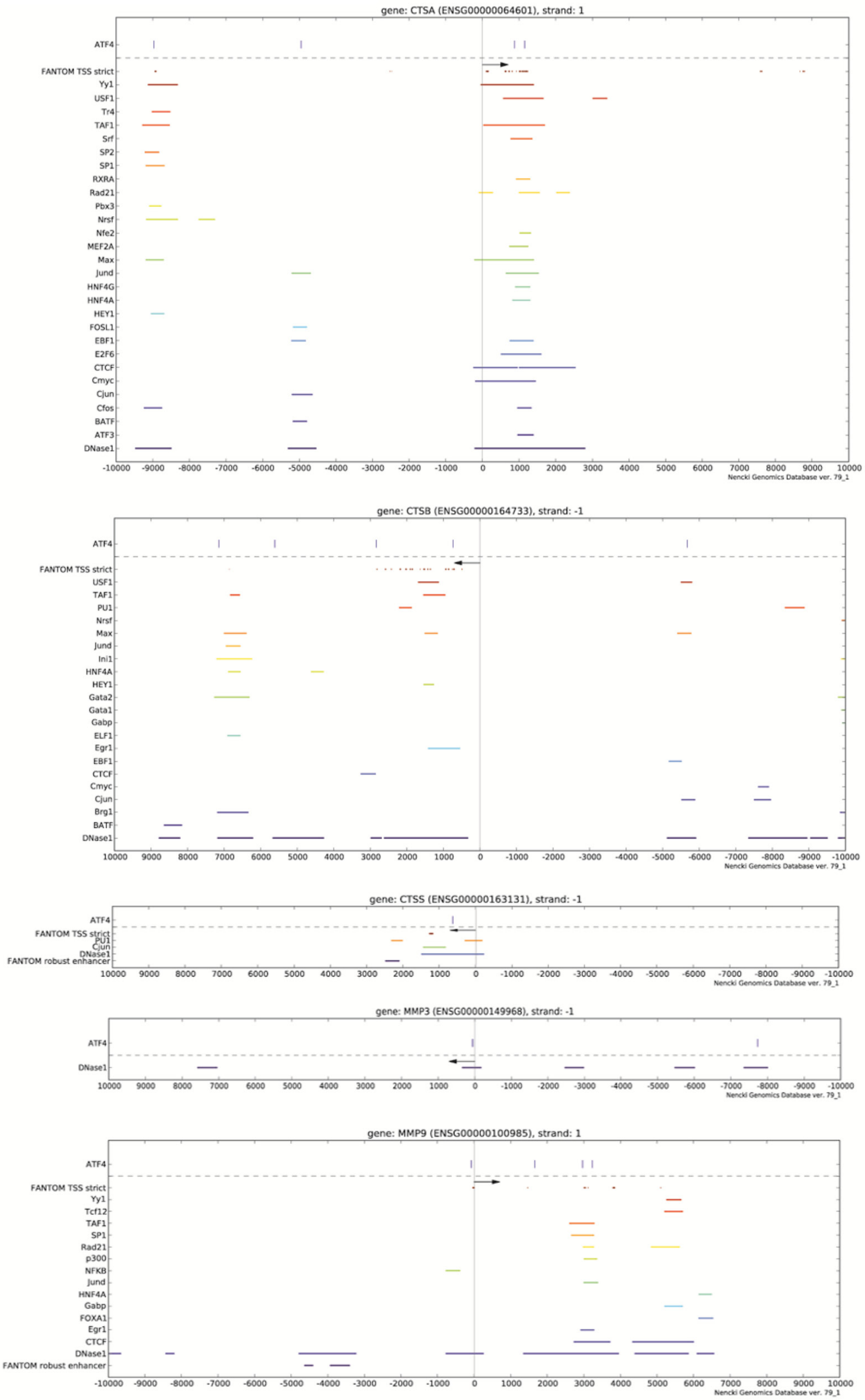

Supplementary Figure S3: Representation of known expression regulatory motifs in selected genes – outcomes of *in silico* analysis using the Nencki Genomics Database.

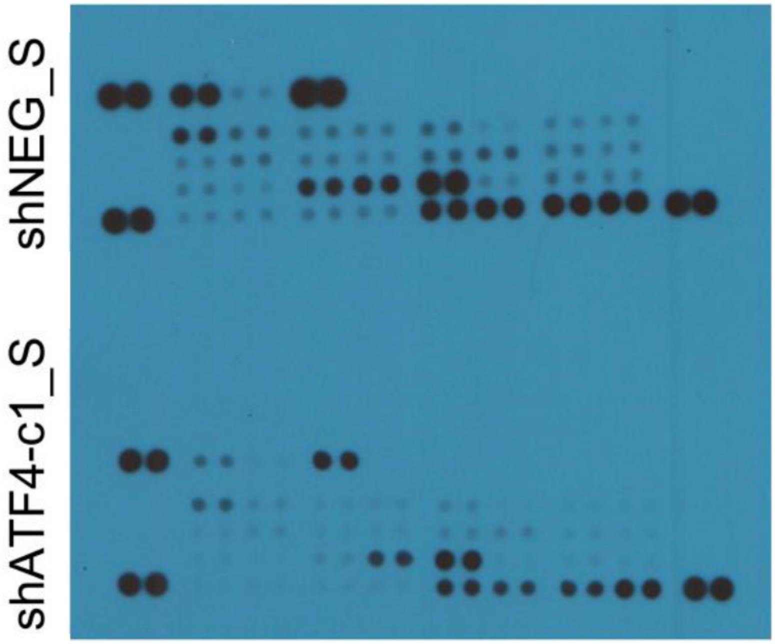

Supplementary Figure S4: Human Protease Array Scan pictures of membranes incubated in conditioned medium by K562 cells transduced with shNEG or shATF4-c1 (shNEG_S and shATF4-c1_S, respectively).

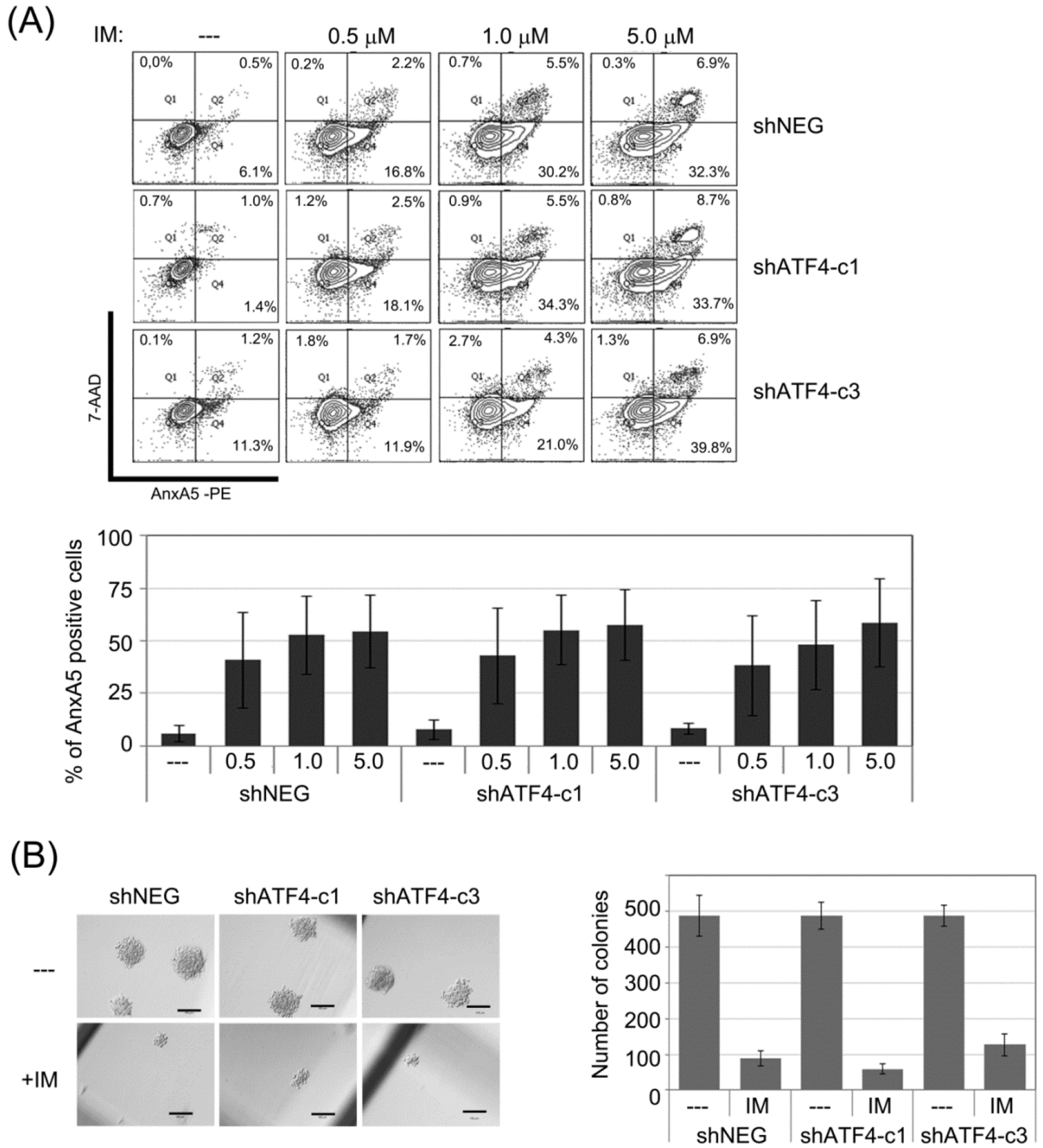

Supplementary Figure S5: Influence of ATF4 knockdown on the sensitivity of K562wt cells to imatinib. Cell death rate of K562wt cells transduced with shRNA control (shNEG) or targeting ATF4 (shATF4-c1 and –c3) **A.** untreated (---) or treated with imatinib (IM) at 0.5, 1.0 or 5.0 µM concentration for 36h. Apoptosis level checked by AnxA5-PE and 7-AAD staining followed by FACS analysis. Typical scatter plots for each culture condition are shown (upper panel), with 7-AAD (y-axis) plotted against AnxA5-PE (x-axis) signal intensity; % of total cell population in each quadrant is given. Graphs in the lower panel show the percentage of dead cells (all AnxA5 positive cells included) in the population as mean ± SEM for n=3 independent experiments. Untreated cells (---) and final µM imatinib concentration indicated on X-axis. **B.** Verification of clonogenic potential by K562 cells expressing shATF4 or shNEG after 1 µM imatinib treatment for 96h. Imatinib added in two doses at 0h and 48h time points. For colony formation assay 1000 of live cells were seeded in MethoCult medium (#04230, StemCells) supplemented with 10% serum (final concentration) and number of colonies formed after 7 days was counted. Images of colonies formed by untreated (---) or imatinib treated (+IM) cells from the light microscope are in the left panel. Scale bar = 100 µm. Graphs in the right panel show the mean number of colonies formed ± SEM for n=3 independent experiments.

Supplementary Table S1: List of proteins with decreased secretion upon reduced eIF2α phosphorylation in K562 cells.

See Supplementary File 1

Supplementary Table S2: Primers used for real-time PCR analysis

Target	GeneBank accession	Forward primer 5'-3'	Reverse primer 5'-3'
18S rRNA	M10098	GTAACCCGTTGAACCCCATT	CCATCCAATCGGTAGTAGCG
ATF4	NM_182810	CTTACGTTGCCATGATCCCT	GAGAACACCTGGAGATGGGA
CTSA	NM_001127695	TGGTCTACTTTGCCTACTACCAT	CACACGGGGCATAGAGATTG
CTSB	NM_147783	AGAGTTATGTTTACCGAGGACCT	GATGCAGATCCGGTCAGAGA
CTSS	NM_001199739	TGACAACGGCTTTCCAGTACA	GGCAGCACGATATTTTGAGTCAT
MMP-3	NM_002422	CAGTTTGCTCAGCCTATCCA	TCACATCTTTTTCGAGGTCGT
MMP-9	NM_004994	GGGACGCAGACATCGTCATC	TCGTCATCGTCGAAATGGGC
