# Supplementary material for: Increased phosphorylation of eIF2α in chronic myeloid leukemia cells stimulates secretion of matrix modifying enzymes
Source: Oncotarget. 2016 Oct 27;7(48):79706–21. doi: 10.18632/oncotarget.12941 (PMC5346746; doi:10.18632/oncotarget.12941)
Supplement: Supplementary file 2 [file oncotarget-07-79706-s002.docx]

**Table 1S.**

**List of proteins with decreased secretion upon reduced eIF2α phosphorylation in K562 cells.**

| **Protein Names** | **Protein IDs** | **Sequence coverage (%)** | **Ratio H/L Normalized (K562mut/wt)** | **Ratio Significance B** |
| --- | --- | --- | --- | --- |
| Tissue-type plasminogen activator | P00750 | 15.7 | -2.54 | 1.4E-13 |
| Cathepsin L1 | P07711 | 49.5 | -1.57 | 1.7E-10 |
| Sulfhydryl oxidase 1 | O00391 | 24.5 | -1.53 | 5.2E-05 |
| Lysosomal thioesterase PPT2 | Q9UMR5 | 10.6 | -1.45 | 1.9E-05 |
| Serpin E1 | P05121 | 25.6 | -1.38 | 2.5E-04 |
| Tartrate-resistant type 5 acid phosphatase | P13686 | 15.7 | -1.35 | 6.9E-05 |
| Alpha-N-acetylgalactosaminidase | P17050 | 11.7 | -1.34 | 7.6E-05 |
| Sialidase-1 | Q99519 | 34.5 | -1.34 | 6.0E-08 |
| ADAM10 | O14672 | 23.8 | -1.32 | 4.7E-04 |
| Beta-galactosidase | P16278 | 21.4 | -1.32 | 4.8E-04 |
| Cathepsin H | P09668 | 61.8 | -1.31 | 1.2E-07 |
| Prosaposin | P07602 | 75.0 | -1.26 | 3.7E-07 |
| Gamma-glutamyl hydrolase | Q92820 | 39.9 | -1.20 | 1.2E-06 |
| Proline carboxypeptidase | P42785 | 26.6 | -1.20 | 1.5E-03 |
| Beta-hexosaminidase subunit alpha | P06865 | 16.1 | -1.20 | 1.3E-06 |
| Procollagen-lysine,2-oxoglutarate 5-dioxygenase 1 | Q02809 | 31.4 | -1.19 | 1.6E-03 |
| Cathepsin C | P53634 | 57.2 | -1.19 | 1.5E-06 |
| Cathepsin B | P07858 | 57.8 | -1.18 | 1.8E-06 |
| Alpha-GalNAcT Exostosin-like 2 | Q9UBQ6 | 34.8 | -1.14 | 2.6E-03 |
| N-acetylgalactosaminyltransferase 7 | Q86SF2 | 12.2 | -1.14 | 7.3E-04 |
| Cathepsin X/Z/P | Q9UBR2 | 60.1 | -1.11 | 7.1E-06 |
| Serine/threonine-protein phosphatase 2A activator | Q15257 | 26,8 | -1.09 | 1.2E-03 |
| Tripeptidyl-peptidase I | O14773 | 44.0 | -1.07 | 1.7E-05 |
| Proprotein convertase 9 | Q8NBP7 | 63.4 | -1.05 | 2.2E-05 |
| Carbonic anhydrase 1 | P00915 | 44.8 | -1.03 | 2.1E-03 |
| Gamma-interferon-inducible lysosomal thiol reductase | P13284 | 28.0 | -1.03 | 2.2E-03 |
| Di-N-acetylchitobiase | Q01459 | 16.4 | -1.01 | 2.7E-03 |
| Palmitoyl-protein thioesterase 1 | P50897 | 61.4 | -0.95 | 1.2E-04 |
| Cathepsin D | P07339 | 64.6 | -0.87 | 4.5E-04 |
| Man(9)-alpha-mannosidase | P33908 | 36.4 | -0.84 | 7.1E-04 |
| Lysozyme C | P61626 | 59.5 | -0.81 | 1.1E-03 |
| Beta-hexosaminidase subunit beta | P07686 | 40.1 | -0.78 | 1.7E-03 |

Presented are the proteins identified by SILAC LC-MS/MS analysis, which corresponding genes were assigned to possess catalytic activity by Gene Ontology Biological Processes database. Results from MaxQuant analysis (performed as described in Cox J and Mann M, 2008 *Nat Biotechnol*, 26:1367-72) of 3 independent experiments are presented.

Legend: Protein Names - UniProtKB/Swiss-Prot names; Protein IDs - UniProtKB/Swiss-Prot database unique identifier; Sequence coverage – gives % of protein sequence covered by identifying peptides; Ratio H/L Normalized - normalized protein group ratio: median of total peptide ratio population log2; H – K562mut; L-K562wt; Significance B – P-value for detection of significant outlier ratio where protein ratio is bin of peptides intensities.
